# The prevalence of human herpesvirus 8 in normal, premalignant, and malignant cervical samples of Iranian women

**DOI:** 10.1186/s12985-021-01614-z

**Published:** 2021-07-10

**Authors:** Sara Chavoshpour-Mamaghani, Zabihollah Shoja, Yaghoub Mollaei-Kandelous, Kimia Sharifian, Somayeh Jalilvand

**Affiliations:** 1 Department of Virology, School of Public Health, Tehran University of Medical Sciences, Tehran, 14155 Iran; 2 Department of Virology, Pasteur Institute of Iran, Tehran, Iran

**Keywords:** Cervical cancer, Human papillomavirus, Human herpesvirus 8, Genotypes

## Abstract

**Background:**

Regard to this fact that the main transmission route of HPV and HHV-8 is via sexual activity, it is reasonable to speculate that coinfection of HPV and HHV-8 may have been played an important role in the development of cervical cancer. The aim of this study was to estimate the prevalence of HHV-8 and the frequency of HPV and HHV-8 coinfection in cervical samples of patients with cervical cancer and healthy individuals.

**Methods:**

In total, 364 samples from 61 patients with cervical cancer, 124 women with premalignant lesions, and 179 healthy individuals were investigated by nested-PCR.

**Results:**

The frequency of HHV-8 was found to be 22.9%, 17.7%, and 14.5% in cervical cancer, premalignant lesions, and normal specimens, respectively (*P* = 0.308). The overall prevalence of coinfection between HHV-8 and HPV was shown to be 16.2%. The HPV prevalence was higher in HHV-8 positive samples than HHV-8 negative specimens in all three studied groups and this difference was reached a statistically significant level (*P* = 0.002). However, no significant differences were found between HHV-8 positivity and HPV genotypes (*P* = 0.08).

**Conclusions:**

Our results showed the higher rate of HHV-8 genome detection in cervical cancer group than control group. However, future studies with larger sample sizes and evaluation of expression of HHV-8 proteins are warranted.

## Introduction

Cervical cancer is considered as the fourth most common cancer with an estimated 604,000 new cases and 342,000 new deaths worldwide in 2020 [1, 2]. The age-standardized incidence rate and mortality rate of cervical cancer in Iran are estimated to be 3.5 and 1.2 per 100,000 females, respectively [3].

Human papillomavirus (HPV) is the most prevalent viral sexually transmitted infection and is well-known as the etiological agent of cervical cancer [4]. Although HPV infections usually clear up within 6 months up to 2 years after acquisition, a small proportion of infections with certain HPV types, designated as high-risk HPV types (HPV 16, 18, 31, 33, 35, 39, 45, 51, 52, 56, 58, 59, 68 and 73), can persist and consequently progress to cervical cancer. Among 12 high-risk HPV types, HPV 16 and 18 are leading cause of cervical cancer and almost 73% of cases are attributed to these two types [4–7]. While HPV infection is necessary to the development of cervical cancer, it is not sufficient and several risk factors including environmental (e.g. number of lifetime sexual partners, oral contraceptive pills, smoking, and infectious agents) and genetic factors are involved in this regard [3, 8, 9]. It is suggested that infection with certain viruses (e.g. herpesviruses and human immunodeficiency virus), bacteria (e.g. Chlamydia trachomatis), fungi, and parasites can increase the risk of cervical cancer [10–14].

Human herpesvirus 8 (HHV-8) has been documented to be the etiological agent of Kaposi’s sarcoma and two kind of lymphomas, including primary effusion lymphoma and Multicentric Castleman disease [15, 16]. HHV-8 establishes life-long latent infection in host cells with the limited expression of several viral genes. HHV-8 can be transmitted through oral, vaginal, and anal sex. It is shown that HHV-8 genome can be detected in saliva, semen, and female genital tract [17, 18].

Regard to this fact that the main transmission route of HPV and HHV-8 is via sexual activity, it is reasonable to speculate that coinfection of HPV and HHV-8 may have been played an important role in development of cervical cancer. To our knowledge, this is the first report of investigating the prevalence of HHV-8 in normal, premalignant, and malignant cervical samples among Iranian women. There are also few studies according to the HHV-8 prevalence in patients with cervical cancer in the world [19]. The aim of this study was to estimate the prevalence of HHV-8 and the frequency of HPV and HHV-8 coinfection in cervical samples of patients with premalignant and malignant lesions as well as healthy individuals.

## Materials and methods

### Samples

To investigate the prevalence of HHV-8, a case–control study was designed. For the purpose of this study, 185 case samples (61 patients with invasive cervical cancer and 124 women with premalignant lesions) and 179 healthy individuals (negative pap-test result) were included. Regard to specimen types, all of 61 samples of patients with invasive cervical cancer and 53 samples of women with premalignant lesions were formalin-fixed paraffin-embedded tissues (FFPE) that were collected from Immam-Khomeini hospital in Tehran and Al-Zahra hospital in West Azarbayzan from 2017 to 2019. Seventy-one samples of women with premalignant lesions and all of 179 normal samples were Thin-Prep Pap Test specimens were obtained from three referral laboratories in Tehran from 2017 to 2019. Although the status of HPV infection and HPV genotypes in 114 FFPE specimens was unknown, they were previously characterized for 250 Thin-Prep Pap Test specimens. The mean age ± SD was 54.1 ± 13.4, 36.1 ± 9.4, and 36.6 ± 11.5 in women with cervical cancer, premalignant lesions, and normal cervix, respectively. All study participants gave informed consent to the study which was approved by the Local Research Ethics Committee (approval no: IR.TUMS.SPH.REC.1399.298) of Tehran University of Medical Sciences.

### DNA extraction

Genomic DNAs from Thin-Prep Pap Test specimens were extracted using a High Pure Viral Nucleic Acid Kit (Roche Diagnostics GmbH, Berlin, Germany) according to the manufacturer’s instruction. Extraction of DNA from FFPE specimens was achieved according to previous published procedure [20]. Briefly, a 10-μm section was manually sectioned and collected in a sterile tube for PCR analysis. To prevent possible cross-contamination between specimens, both blades and gloves were changed before cutting next block. Each section of tissue biopsies was treated twice with xylene to remove paraffin and twice with absolute ethanol to remove organic solvents. Tissue samples were then digested by lysis buffer (10 mM Tris–HCl pH 7.6, 5 mM EDTA, 150 mM NaCl, 1% SDS) containing 150 μg per ml Proteinase K (56 °C for 3 h), followed by DNA purification by phenol and phenol–chloroform–isoamyl alcohol (25:24:1) extraction and ethanol precipitation in 0.3 M sodium acetate (pH 4.6). The integrity of extracted DNA from FFPE samples were evaluated using amplification of a 268 bp fragment of beta-globin gene [21]. Following DNA quality assays, it is confirmed that all of 114 FFPE samples were suitable for HHV-8 detection.

### PCR amplification of L1 gene of human papillomavirus (HPV) and genotyping

HPV DNA detection in all FFPE samples (114 samples) was carried out by nested-PCR using MY09/MY11 primer pair (outer primers) and GP5 + /GP6 + primer pair (inner primers) to obtain almost a 150 bp fragment of HPV L1 gene [22]. The PCR reactions was performed in a 50 μL reaction mixture including 100–200 ng of DNA template, 2.5 mM of MgCl_2_, 10 pmol of each primer, 50 mM of each dNTP, and 2 U of Taq DNA polymerase. PCR amplification cycles were as follow for the first and the second rounds: an initial 3-min denaturation at 95 °C, followed by 35 cycles of 95 °C for 30 s, 50 °C for 45 s, and 72 °C for 1 min (first round) and 35 cycles of 95 °C for 30 s, 52 °C for 40 s and 72 °C for 45 s (second round), and a final elongation for 5 min at 72 °C. A reaction mixture lack of DNA template, as a negative control, was included in each run of PCR. The PCR products were run on a 2% agarose gel.

The PCR amplification products were sequenced using BigDye® Terminator v3.1 Cycle Sequencing Kit and a 3130 Genetic Analyzer Automated Sequencer as specified by Applied Biosystems manuals (Foster City, CA). Nucleotide sequences were edited with Bioedit software and converted to FASTA format. Then, sequences compared to other HPV types using the Blast server (http://www.ncbi.nlm.nih.gov/blast/).

### PCR amplification of ORF26 gene of human herpesvirus 8 (HHV-8)

ORF26 is a conserved gene and uses to detect of HHV-8 in clinical samples [15]. HHV-8 ORF26 was amplified using nested-PCR to obtain a 172 bp amplicon size. The sequences of primers that used in this study were 5’-AGCCGAAAGGATTCCACCATT-3’and 5’-TCCGTGTTGTCTACGTCCAGA-3’for the first round and 5’-GTGCTCGAATCCAACGGATT-3’ and 5’-ATGACACATTGGTGGTATATAG-3’ for the second round [23]. The PCR reaction was achieved in a 50 μl reaction mixture including 100–200 ng of DNA template, 1.5 mM MgCl_2_, 50 μM of each dNTP, 10 pmol of each primer, and 1.5 U of Taq DNA polymerase. PCR amplification cycles were as follow for the first and the second rounds: 35 cycles of 95 °C for 30 s, 55 °C for 40 s and 72 °C for 50 s and 35 cycles of 95 °C for 30 s, 52 °C for 40 s and 72 °C for 45 s, respectively. A reaction mixture lack of DNA template, as a negative control, was included in each run of PCR. The PCR products were run on a 2% agarose gel.

### Statistical analysis

Statistical analysis was performed by EPI Info 7 Statistical Analysis System Software (Atlanta, GA, USA) using the X^2^ test or the Fisher exact test. When the P-values were less than 0.05, they were considered statistically significant.

## Results

The prevalence of HHV-8 in 364 cervical samples from 61 patients with cervical cancer, 124 women with premalignant lesions, and 179 healthy individuals were investigated in this study.

The mean age ± SD was 54.1 ± 13.4, 36.1 ± 9.4, and 36.6 ± 11.5 in women with cervical cancer, premalignant lesions, and normal cervix, respectively.

The frequency of HHV-8 was found to be 22.9%, 17.7%, and 14.5% in cervical cancer, premalignant lesions, and normal specimens, respectively. Although the prevalence of HHV-8 was higher among cervical cancer patients compared to premalignant lesions and normal specimens, the difference was not statistically significant (*P* = 0.308) (Table 1). Among women with cervical cancer, no significant differences were found between the prevalence of HHV-8 and cancer types as it was found in 24% of squamous cell carcinoma (SCC) and 18.2% of adenocarcinoma (AD) cases (*P* = 0.98). Interestingly, no statistically significant differences were found between the prevalence of HHV-8 and sample types (*P* = 0.78) as HHV-8 was detected in 15.8% and 17.6% of FFPE and Thin-prep pap test samples, respectively (Table 1).

**Table 1:** The prevalence of human herpesvirus 8 (HHV-8) in uterine cervical samples, stratified by several factors

Total	HHV-8 status			*p*-value
	Positive N (%)	Negative N (%)	Total N (%)	
	62 (17)	302 (83)	364 (100)	
*Samples*				
Normal	26 (14.5)	153 (85.5)	179 (100)	
Premalignant	22 (17.7)	102 (82.3)	124 (100)	0.308
Malignant	14 (22.9)	47 (77.1)	61 (100)	
Total	62 (17.0)	302 (83.0)	364 (100)	
*Sample types*				
FFPE	18 (15.8)	96 (84.2)	114 (100)	
Thin-prep	44 (17.6)	206 (82.4)	250 (100)	0.78
Total	62 (17)	302 (83)	364 (100)	
*HPV infection*				
Positive	59 (20.3)	233 (79.7)	292 (100)	
Negative	3 (4.0)	69 (96.0)	72 (100)	**0.002**
Total	62 (17)	302 (83)	364 (100)	
*HPV genotypes*				
HPV 16/18	19 (15.7)	102 (84.3)	121 (100)	
High-risk HPVs (non-16/18)	40 (23.4)	131 (76.6)	171 (100)	0.1
Total	59 (20.3)	233 (79.7)	292 (100)	
*Cancer types*				
SCC	12 (24.0)	38 (76.0%)	50 (100)	
AC	2 (18.2)	9 (81.8)	11 (100)	0.98
Total	14 (22.9)	47 (77.1)	61 (100)	
*Age group*				
20–30	7 (11.1)	52 (88.1)	59 (100)	
30–40	8 (10.8)	66 (89.2)	74 (100)	
40–50	5 (10.7)	42 (89.3)	47 (100)	0.55
> 50	10 (18.5)	44 (81.5)	54 (100)	
Total	30 (12.8)	204 (87.2)	234 (100)	

*P*-values less than 0.05 are indicated in bold font

SCC: Squamous cell carcinoma and AD: Adenocarcinoma; FFPE: Formalin-fixed paraffin-embedded tissue

As shown in Table 1, the prevalence of HHV-8 was different regard to HPV infection status as higher rate of HHV-8 infection was observed among HPV positive samples (20.3%) than HPV negative samples (4.0%) and this difference was reached a statistically significant level (*P* = 0.002).

**Fig. 1:**
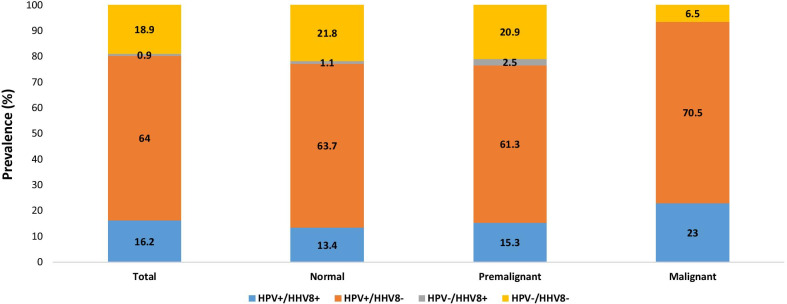
The frequency of human herpesvirus-8 (HHV-8) and human papillomavirus (HPV) in malignant, premalignant, and normal groups

As presented in Fig. 1, the overall prevalence of coinfection between HHV-8 and HPV was shown to be 16.2%. The HPV prevalence in three studied groups was different regard to HHV-8 infection status: higher rate of HPV infection was found among women with cervical cancer (23%) compared to premalignant and normal groups that coinfection was shown in 15.3% and 13.4%, respectively (Fig. 1). Among HHV-8 positive samples, the frequency of HPV was 92.3%, 86.3%, and 100% in cervical cancer, premalignant lesions, and normal samples, respectively (Table 2).

**Table 2:** The analysis between human herpesvirus 8 (HHV-8) positivity and age group, human papillomavirus infection (HPV), and HPV genotypes in three studied groups

	Studies groups			
	Normal N (%)	Premalignant N (%)	Malignant N (%)	
*HPV infection*				
HPV positive	24 (92.3)	19 (86.3)	14 (100)	
HPV Negative	2 (7.7)	3 (13.7)	0 (0.0)	0.34
Total	26 (100)	22 (100)	14 (100)	
*HPV genotypes*				
HPV 16/18	5 (20.8)	3 (15.8)	11 (78.6)	
High-risk HPVs (non-16/18)	19 (79.2)	16 (84.2)	3 (21.4)	**0.0001**
Total	24 (100)	19 (100)	14 (100)	
*Age groups (yr.)*				
> 40	18 (69.2)	12 (54.5)	3 (21.4)	
< 40	8 (30.8)	10 (45.5)	11 (78.6)	**0.015**
Total	26 (100)	22 (100)	14 (100)	

*P*-values less than 0.05 are indicated in bold font

Regard to HPV genotypes, the frequency of HHV-8 was 15.7% and 23.7% in samples coinfected with HPV 16/18 and high-risk non-16/18 HPV types (HPV 31, 33, 35, 39, 45, 51, 52, 56, 58, and 59), respectively. However, no significant differences were found between HHV-8 positivity and HPV genotypes (*P* = 0.08) (Table 1). Interestingly, among HHV-8 positive samples, a statistically significant difference was found regard to HPV genotypes in three studied groups (*P* = 0.0001) as coinfection with HPV 16/18 was more common in cervical cancer samples (78.6%) than premalignant (15.8%) and normal (20.8%) groups (Table 2). In other words, high-risk non-16/18 HPV types was more frequent in normal (79.2%) and premalignant (84.2%) groups compared to cervical cancer group (21.4%).

As shown in Table 1, although the prevalence of HHV-8 was higher among women more than 50 years-old (18.5%) in comparison to other age groups, no statistically significant differences was found (*P* = 0.55). However, in three studied groups that were HHV-8 positive, a meaningfully significant difference was found regard to age groups (*P* = 0.015, Table 2). As shown in Table 2, whereas the most of normal (69.2%) and premalignant (54.5%) samples were in age group of < 40 years old, the most of malignant samples (78.6%) were found in group > 40 years old.

## Discussion

There are few studies have been investigated the role of coinfection between HPV and HHV-8 or their interaction in cervical cancer. In this study, the prevalence of HHV-8 and the frequency of HPV/HHV-8 coinfection were evaluated in uterine cervical samples. HHV-8 genome was detected in 22.9%, 17.7%, and 14.5% of cervical cancer, premalignant lesions, and normal specimens, respectively. In agreement with our results, in a study from China, HHV-8 genome had been found in 4.5% and 10% of cervical intraepithelial neoplasia (CIN) II–III and cervical cancer, respectively. HHV-8 DNA had been also detected in 9.2% and 14.3% of normal and CIN I samples, respectively [19]. HHV-8 infection was found in 16% and 8% of Spanish sex workers and general population, respectively and the odds ratio was found to be 2.2 [24]. HHV-8 DNA was also found in 3.2% and 0% of male and female genital brushing in Brazil, respectively [25]. However, HHV-8 DNA was not detected in any normal cervical secretions of Swedish women samples [26]. The discrepancy between these studies can be due to this fact that HHV-8 is shed only intermittently in infected individuals [26].

It is suggested in regions that the prevalence of HHV-8 is medium or low, oral shedding and heterosexual contacts are potential pathways for HHV-8 transmission [24, 27]. A study from Kenia in HIV-1 seropositive and seronegative individuals had been revealed that a 2.1-fold-increased odds of HHV-8 shedding from genital site was occurred among HIV-1 seropositive than seronegative women. They were found HHV-8 shedding in the genital tract is unrelated to HIV-1 viral load and CD4 count. Consequently, they were suggested that the underlying mechanism of HHV-8 shedding from genital mucosal site may be independent of cellular immunity as measured by CD4 count [28]. In a previous study from Iran, HHV-8 DNA was detected in 13.6% of intravenous drug users and 3.6% of general population on peripheral blood mononuclear cell (PBMC) samples. Interestingly, in both groups, the frequency of HHV-8 was higher among women than men although the difference was not statistically significant [23]. The results of one study had been revealed that the genome of HHV-8 was detected in 2% and 1% of the cervical samples of the prostitutes women and the general population, respectively. However, all PBMC samples of these women were negative [24]. It is suggested that HHV-8 infection was higher among women with presumable high-risk behavior for acquiring sexually transmitted diseases (i.e., early age at first sexual intercourse and HPV cervical infection) [24].

A statistically significant difference was observed between the frequency of HHV-8 and HPV infection as the prevalence of HHV-8 was remarkably higher among HPV positive samples than HPV negative samples. In accordance to our data, a study from Spain was shown that HHV-8 was more common among HPV DNA-positive women (odds ratio = 2.5) and among women with an early age at first sexual intercourse (Odds ratio = 2.7) [24]. However, a study from china was found that the frequency of HHV-8 among HPV-positive and HPV-negative groups were similar (9.4% vs. 8.0%) [19].

In this study, totally no significant differences were found between HHV-8 positivity and HPV genotypes. However, the prevalence of HPV genotypes was meaningfully different between cervical cancer patients and premalignant or normal subjects and this difference was statistically significant as HPV 16/18 was more prevalent in cervical cancer specimens (78.6%) rather than normal (20.8%) and premalignant samples (14.3%).

HHV-8 infection synergistically with HPV can contribute to cervical carcinogenesis through several mechanisms. It is shown that HHV-8 coinfection in SiHa cell line could be promoted the increased expression levels of several inflammatory factors, including interleukin 6 (IL-6), chemokine (C-X-C motif) ligand 1 (CXCL1), chemokine (C–C motif) ligand 5 (CCL5), interleukin 8 (IL-8), macrophage migration inhibitory factor (MIF), and plasminogen activator inhibitor-1 (PAI-1) [29, 30]. Indeed, IL-6 has been shown to stimulate cervical tumor growth through vascular endothelial growth factor (VEGF)-dependent angiogenesis [31]. Moreover, CXCL1 is a main factor in the development of cervical cancer as the increased level of CXCL1 was reported in serum of patients with cervical cancer in comparison to premalignant and healthy controls [32]. Regard to this fact that chronic inflammation is a one of the hallmarks of cancer, it is tempting to speculate HHV-8 coinfection may play an important role to support cervical carcinogenesis via providing chronic inflammation. Interestingly, HHV-8 encodes v-IL6 in lytic phase [33, 34] that can also be to enhance and promote carcinogenesis of cervical cancer. Moreover, HHV-8 encoded lytic protein, RTA (Replication and transcription activator), can bind to at least three sites within HPV 16 genome, including positions 7522-7333 and 7590-7603 in long control region (LCR) and 333-344 in E6 coding gene and lead to a substantial upregulation of E7 transcription [35]. HPV E7 and E6 viral oncoproteins play central role in driving the cells toward tumorigenesis. These two proteins can trigger all of the hallmarks of cancer cell [36, 37]. Taken together coinfection of HPV/HHV-8 could be played direct and indirect roles in cervical cancer carcinogenesis.

In three studied groups that were HHV-8 positive, a meaningfully significant difference was found regard to age groups (*P* = 0.015). While the most of normal (69.2%) and premalignant (54.5%) samples were in age group of < 40 years old, the most of malignant samples (78.6%) were found in group > 40 years old. These results are reasonable as the peak of cervical cancer incidence is shown to be in 55–65 year-old women in Iran [38]. Regard to these facts that more reactivation of HHV-8 can take place with increasing age and the classic Kaposi’s sarcoma is more often happened in elderly people, it is also possible HHV-8 reactivated more common in women with cervical cancer due to aging-associated immunosuppression [39, 40].

The most important limitations of this study were found to be the relatively small sample size.

## Conclusions

Our results showed higher rate of HHV-8 genome detection in cervical cancer group than premalignant and control groups. Moreover, our finding supports the likelihood of HHV-8 transmission through sexual route in Iranian population. However, future studies with larger sample sizes and evaluation of expression of HHV-8 proteins on cervical samples are warranted. It is also recommend that the frequency of HHV-8 shedding will be determined in saliva of all age groups to clarify the transmission routes of HHV-8 in Iran.

## Data Availability

Data available within the article.

## Abbreviations

HHV-8: Human herpesvirus 8
HPV: Human papillomavirus
FFPE: Formalin-fixed paraffin-embedded tissues
SCC: Squamous cell carcinoma
AD: Adenocarcinoma
CIN: Cervical intraepithelial neoplasia
